# Quality Evaluation of Ready-to-Use Various Brown Rice (*Oryza sativa*) Powder Using Extrusion Process

**DOI:** 10.3390/foods14223948

**Published:** 2025-11-18

**Authors:** Jittimon Wongsa, Witoon Khawsuk, Tistaya Semangeon, Prateep Oupkaew, Karthikeyan Venkatachalam, Parinyaporn Nuurai, Narin Charoenphun

**Affiliations:** 1 Faculty of Industrial Technology and Management, King Mongkut’s University of Technology North Bangkok (Prachinburi Campus), Prachinburi 25230, Thailand; jittimon.w@itm.kmutnb.ac.th; 2 Faculty of Allied Health Sciences, Burapha University, Chonburi 20131, Thailand; witoon@go.buu.ac.th (W.K.); tistaya@go.buu.ac.th (T.S.); 3 Agricultural Innovation, Faculty of Agricultural Technology, Burapha University, Sa Kaeo Campus, Sa Kaeo 27160, Thailand; prateep_o@buu.ac.th; 4 Faculty of Innovative Agriculture, Fisheries and Food, Prince of Songkla University, Surat Thani Campus, Makham Tia, Mueang, Surat Thani 84000, Thailand; karthikeyan.v@psu.ac.th; 5 Faculty of Science and Arts, Burapha University, Chanthaburi Campus, Chanthaburi 22170, Thailand

**Keywords:** brown rice, extrusion process, anthocyanin, antioxidant, calcium oxalate crystal formation

## Abstract

Extrusion is a method for preparing ready-to-use rice powders that can help reduce cooking time. This study aimed to investigate the effects of extrusion on the physicochemical properties, antioxidant, and anti-calcium oxalate crystal formation activities of rice powder from eight brown rice varieties. The results showed that all eight rice samples had moisture contents of 6.55–7.32% and aw values of 0.43–0.49. Glutinous rice has a higher water absorption index and swelling power than non-glutinous rice. The analysis of the aroma values using PCA and k-means revealed that the samples could be divided into two major groups based on their rice aroma, group 1 (RD43, Hom Mali 105, and Hom Mali Daeng) and group 2 (Khiaw Ngoo, Riceberry, Luem Pua, RD6, and Kum Lanna). RD43, Hom Mali 105, and Hom Mali Daeng had high peak viscosities (102–126 cP) and high breakdown values (51–83 cP), indicating high swelling. Riceberry had low peak viscosity values (27 cP) but high setback viscosity values (26 cP), indicating a strong tendency to retrogradation. In terms of bioactivity, Colored rice varieties contain high levels of total monomeric anthocyanins and phenolic compounds and exhibit strong antioxidant activity. Colored rice and colored glutinous rice exhibited a high percentage inhibition of calcium oxalate crystal formation (40.03–58.71%). This study highlights the importance of extrusion processing methods in preserving the nutritional benefits of different brown rice powders, which can be used in the food industry to enhance the nutritional value of rice products.

## 1. Introduction

Rice (*Oryza sativa*) is one of the most crucial crops for social stability and food security [1]. Asia, Sub-Saharan Africa, and South America are the major regions for rice cultivation. The amount of rice consumed tends to increase rapidly and this could be the result of faster population expansion both urbanization and shifting consumer demands [2]. Each rice strain has various characteristics that are similar or different, including color, size, shape, and appearance. Pigments, such as anthocyanins, are important components that give rice its characteristics, including black, red, and purple colors. Anthocyanins are polyphenols that belong to the flavonoid group. It has nutraceutical properties, antioxidant activity, aids in blood circulation, and slows the aging process. Flavonoids have antioxidant properties. They can protect the body from damage caused by free radicals. High levels of free radicals can lead to oxidative stress, which accelerates disease and the aging process. Flavonoids inhibit cell and deoxyribonucleic acid (DNA) damage caused by free radicals, thus helping to slow aging [3]. Currently, rice is processed into various forms, including rice powder, pre-cooked rice flours, or instant powdered beverage products, which are also interesting products for consumers. This liquid food product is particularly beneficial for individuals experiencing difficulties with mastication, as it allows for effortless swallowing. Consumption of pre-cooked rice flours takes 3–5 min for preparation, but producing normal rice porridge takes an average of 30–60 min [4]. It is easily digested, suitable for the elderly and patients who require easily digestible food, and is an alternative product for consumers who are allergic to wheat gluten. Pre-cooked rice flour or instant powdered beverage is a product obtained by washing rice thoroughly, followed by baking or partial cooking. It is ground into powder that may be flavored with sugar, salt, and other ingredients such as milk and powdered or ground grains before consumption by adding hot water and stirring well [5]. There are many factors that affect the properties of instant pre-cooked rice flours, including the growing environment (temperature and soil conditions), processing method, and chemical composition, especially the proportion of amylose and amylopectin. This affects the physicochemical properties of rice flour and the texture of the final product. The good characteristics of the pre-cooked rice flour must be flaking or powder, dry, not clumped, free from undesirable flavors, natural color, and scent [6].

Processing methods play a crucial role in determining the physicochemical properties of rice-based products. To produce instant or pre-cooked rice flour, various drying and cooking techniques have been applied; however, each has specific limitations. Hot air and drum drying require high temperatures and long processing times, which can lead to nutrient losses and undesirable color changes [7]. Spray drying is unsuitable for brown rice because of its high viscosity [8], while foam-mat drying yields low productivity and is not feasible for continuous production [9]. Freeze drying, though capable of preserving nutrients, involves high operational costs and is impractical for large-scale manufacturing [10]. Extrusion, on the other hand, is a high-temperature short-time (HTST) processing technique widely used in producing instant and pre-cooked rice flours. Compared with conventional methods, extrusion offers several advantages, including continuous operation, precise control of temperature and moisture, and simultaneous mixing, cooking and shaping. This process enhances starch gelatinization, digestibility, and product uniformity, key attributes for high-quality instant rice products [11]. Previous studies have confirmed that extrusion significantly modifies the physicochemical and functional properties of rice flour, reducing cooking time and improving solubility and dispersibility in water [12].

Bioactive phytochemicals in rice and associated biological activities such as antioxidant and antiurolithiasis are effective. In general, bioactive compound is found in brown rice including phenolic acids, flavonoids, other phenolics, dietary fibre, carotenoids, tocols, phytosterols, gamma-oryzanol, and phytic acid [13]. The antioxidant activity in rice is mainly related to bioactive compounds such as flavonoids and phenolic acids. These compounds help neutralize free radicals and reduce oxidative stress, thus providing a variety of health benefits. The main mechanism involves free radical scavenging, where antioxidants such as quercetin and anthocyanins in rice varieties neutralize reactive radicals (ROS), including 2,2-diphenyl-1-picrylhydrazyl (DPPH) and 2,2′-azino-bis(3-ethylbenzothiazoline-6-sulfonic acid) (ABTS). Antioxidants also regulate enzymes such as superoxide dismutase, catalase, and glutathione peroxidase, which help maintain cellular balance and protect against oxidative damage [14]. Other compounds such as phloricin, trilobatin, and myricetin 3-galactoside contribute to the antioxidant properties of some rice varieties, particularly purple and black rice, which are rich in anthocyanins. The total flavonoid and total anthocyanin content of rice is generally measured to assess its antioxidant capacity, with higher amounts indicating stronger antioxidant activity [15]. In terms of antiurolithiasis, brown rice has antiurolithiasis properties because it is high in dietary fiber, which reduces the absorption of calcium oxalate (CaOx), the main component of kidney stones. Brown rice is also rich in magnesium and potassium, which help reduce the concentration of stone-forming substances in the urine, and has antioxidants that reduce inflammation in the kidneys. The myo-inositol extraction from Japanese rice bran inhibits CaOx crystal formation, which has been shown to be effective against urinary tract stones by converting calcium oxalate monohydrate to the more easily excreted dihydrate form [16].

Despite extensive studies on extrusion-cooked rice flours, limited information is available regarding the combined evaluation of antioxidant and anti-CaOx crystal formation activities of pre-cooked brown rice flours from different varieties. Therefore, this study aims to investigate the effects of the extrusion process on the physicochemical properties, antioxidant capacity, and calcium oxalate crystal formation inhibition of pre-cooked brown rice flours from eight brown rice (*Oryza sativa*) varieties. The rationale for selecting the eight brown rice varieties used in this study is that they are popular in Thailand and are commercially available. There were four groups of samples: group A: white rice ((R1) RD43 and (R2) Hom Mali 105), group B: white glutinous rice ((R3) RD6 and (R4) Khiaw Ngoo), group C: colored rice ((R5) Hom Mali Daeng and (R6) Riceberry), and group D: colored glutinous rice ((R7) Leum Pua and (R8) Kum Lanna). This research will provide useful quality information and guidelines for rice processing business operators. Consumers interested in pre-cooked rice powder and those interested in developing further processed food products from rice, including creating added value from brown rice.

## 2. Materials and Methods

### 2.1. Raw Materials

The rice used in the experiment was 8 brown rice varieties as follows: (R1) RD43, (R2) Hom Mali 105, (R3) RD6, (R4) Khiaw Ngoo, (R5) Hom Mali Daeng, (R6) Riceberry, (R7) Leum Pua, and (R8) Kum Lanna. The rice varieties were purchased from farmers in Chiang Rai, Sa Kaeo and Prachinburi provinces and stored in sealed containers. All samples were indica rice, which was popular in tropical regions. The grains were long and slender. It was planted in the rainy season in Thailand from May to October and harvested in the winter around the end of November to December. The harvest period was about 140–150 days. The chemicals and reagents used in this study were of analytical grade (purity) and these include ethanol (99.8%), acetone (99.9%), potassium chloride (99.8%), sodium acetate (99.5%), gallic acid (99.5%), Folin–Ciocalteu reagent (99.9%), sodium carbonate (99.8%), DPPH (2,2-diphenyl-1-picrylhydrazyl) (99.0%), ascorbic acid (98.0%), ferric sulfate (99.0%), sodium phosphate (99.8%), potassium ferricyanide (98.0%), trichloroacetic acid (99.0%), ferric chloride (99.0%), calcium chloride (97.0%), sodium oxalate (99.5%), Tris-hydrochloride (99.0%), and sodium chloride (99.7%). The chemicals used in the study were obtained from LOBA Chemie Pvt Ltd. (Mumbai, India) and Sigma Aldrich (Saint Louis, MO, USA).

### 2.2. Proximate Analysis of Brown Rice Grain

The proximate analysis of brown rice grain was investigated including moisture, protein, fat, total carbohydrate, total dietary fiber, ash, reducing sugar, and energy [17].

### 2.3. Rice Powder Preparation

**Figure 1:**
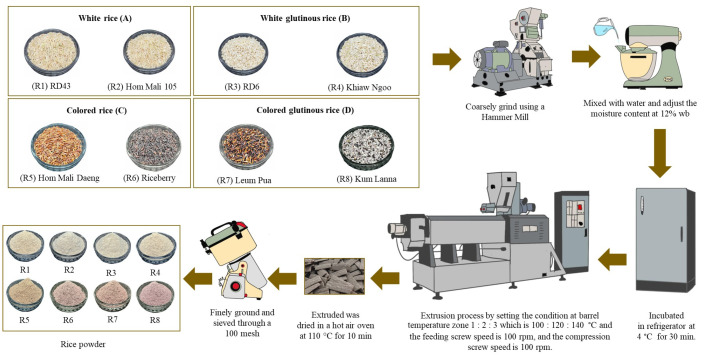
Rice powder preparation.

The eight rice grains samples were coarsely ground using a hammer mill (GmbH, model 5657 HAAN; Retch, Haan, Germany). The moisture content (12% wb) of each type of rice flour was adjusted with water and then mixed using a stand mixer (Kenwood, Havant, UK) for 5 min. The mixture was packed in a plastic bag and incubated in a refrigerator at 4 °C for 30 min before the extrusion process. A single screw laboratory extruder (Brabender, Extruder 19/20 DN, Duisburg, Germany) equipped with a flat sheet die head (15 mm width × 3 mm height) was used in this study. The initial moisture content of the various rice flour materials was adjusted to 12 percent. The barrel temperature zones were set to 100 °C, 120 °C and 140 °C, and both the feeding and compression screw speeds were fixed at 100 rpm. The feed rate was maintained at 6.5–7 kg/h. The eight samples of Extruded rice were cut into small pieces and dried in a hot air oven at 110 °C for 10 min. Extruded rice samples were finely ground using a fine grinder (500 g Multi-Function Disintegrator, WF-10B, Taipei, Taiwan) and sieved through a 100 mesh. The extruded rice powder was stored in sealed aluminum foil bags (Figure 1).

### 2.4. Physicochemical Analysis of Rice Powder

#### 2.4.1. Color

Color values were measured using a benchtop colorimeter (Hunter Lab, Color flex, Reston, VA, USA) according to the CIE L* a* b* system. L* value or lightness (0 = black, 100 = white), a* value (+a = red, −a = green), and b* value (+b = yellow, −b = blue) were investigated. All color measurements were performed in five replicates per sample.

#### 2.4.2. Moisture and Water Activity (a_w_)

Moisture content was measured using an infrared food moisture meter (MOC-120H model, SHIMADZU brand, Kyoto, Japan) by placing 5 g of sample into an analysis cup. The quantity of free water was measured using a water activity meter (4TE, Aqualab, Pullman, WA, USA). The sample was placed in an analytical cup and measured at 25 °C. Three replicate measurements were performed for each sample.

#### 2.4.3. Aroma Analysis of Rice Powder

The aroma characteristics of the rice samples were analyzed using an electronic nose (E-nose). Instant powdered beverage products were prepared for aroma analyses. Hot water (100 °C) was added to rice at a ratio of 30 mL hot water to 2.5 g of rice powder. Aroma detection was conducted using an e-nose (MUI Nose 1.1 Portable Electronic Nose, MUI Robotics Co., Ltd., Bangkok, Thailand) at room temperature. The rice powder samples were analyzed for aroma using VDI/VDE 3518 odor-related measurements with an electronic nose. The test conditions were 2.5 g of sample at 25 °C and a flow rate of 1 L/min.

#### 2.4.4. Water Absorption Index (WAI), Water Solubility Index (WSI), and Swelling Power (SP)

WAI, WSI, and SP were determined with some modifications using the method described by Harasym et al. [18]. For the WAI analysis, 2.5 g of the sample was placed in a centrifuge tube of known weight, and 30 mL of distilled water was added. The sample solution was stirred with a glass rod for 30 min and centrifuged at 4500 rpm for 35 min. The clear liquid was poured into a moisture can of a known weight. The centrifuge tube and resulting gel were weighed. In terms of WSI analysis, the moisture contained in the supernatant obtained from the WAI analysis was evaporated to dryness and then placed in a vacuum oven (VD 23, Binder, Tuttlingen, Germany) at 70 °C for 5 h. The moisture can was then placed in a desiccator for 1 h and weighed. The obtained values were used to calculate WAI, WSI, and SP using the following equations:

(1) WAI (g/g dry basis) = (Wg − Ws)/Ws

(2) WSI (g/g dry basis) = (Wsl × 100)/Ws

(3) SP (g/g dry basis) = (Wg − Wsl)/Ws

where Wg is the weight of the gel, Ws is the initial weight of the sample, and Wsl is the weight of solids obtained from the supernatant. The results are expressed as grams of water absorbed per gram of dry basis (d.b.).

#### 2.4.5. Viscosity Analysis

Pasting properties were analyzed using a Rapid Visco Analyzer (RVA-TecMaster, Perten PerkinElmer, Springfield, IL, USA) with some modifications by Wongsaipun et al. [19]. A 2.8 g sample (dry weight) was placed in an RVA canister. Distilled water was added to the RVA canister to achieve a total weight of 23 g. The mixture was thoroughly mixed before beginning the analysis. The analysis was started at 50 °C for 0–1.5 min at 960 rpm for the first 10 s. The speed was then reduced to 160 rpm and maintained throughout the analysis. The temperature was increased to 95 °C for 1.5–5 min, held at 95 °C for 2.5 min, and then lowered to 50 °C and held at 50 °C for 1.5 min. The total testing time was 12.5 min. The results of the RVA viscosity measurement are displayed on a graph with the x-axis representing time and the y-axis representing two axes: one side represents the viscosity in RVUs and the other represents the temperature. The results of the RVA viscosity analysis graph can be interpreted to yield various values that reflect the properties of the starch, as follows: peak time (min) is the time at which maximum viscosity occurs. The pasting temperature is the temperature at which gelatinization begins, and the starch viscosity changes rapidly, as measured by the increase in viscosity. The peak temperature (°C) is the temperature at which the maximum viscosity occurs. The holding strength (RVU) is the lowest viscosity during cooling. Final viscosity (RVU) is the final viscosity in the experiment. The values were calculated as follows:

(4) Breakdown (RVU) = Peak viscosity − Holding strength

(5) Setback form peak (RVU) = Final viscosity − Peak viscosity

(6) Setback form trough (RVU) = Final viscosity − Holding strength

### 2.5. Bioactive Properties of Rice Powder

#### 2.5.1. Preparation of Crude Instant Rice Powder Extracts

Instant rice powder was extracted with 75% ethanol at a dilution of 1:5 for 24 h at room temperature with gentle shaking, protected from light. The extract solution was then filtered and collected. The residue was re-extracted twice with 75% ethanol. The pooled extract solution was then evaporated using a rotary evaporator and dried using a freeze dryer. The extraction yield was calculated, and the extract was stored at −20 °C until use.

#### 2.5.2. Determination of Total Monomeric Anthocyanin Content

The total monomeric anthocyanin content was determined using the pH differential method, as described by Khawsuk et al. [20]. Briefly, crude instant rice powder extracts were prepared as a 10% w/v sample solution using 40% acetone as the solvent. The appropriate sample dilution was determined by adding 0.025 M potassium chloride buffer (pH 1.0). The absorbance of the samples was measured at 510 nm using a spectrophotometer. The appropriate sample dilution was selected from the linear portion of the graph. The test sample was then quantified by adding 0.025 M potassium chloride buffer (pH 1.0) and 0.4 M sodium acetate buffer (pH 4.5) and incubated at room temperature in the dark for 20 min. The absorbance was measured at 510 and 700 nm, respectively, and used for direct calculation according to the following equation:

Monomeric anthocyanin content (mg/L) = (A × MW × DF × 1000)/(ε × l)

where

A = (A_520_ − A_700_) pH 1.0 − (A_520_ − A_700_) pH 4.5;

MW = 449.2 g/mol (cyanidin-3-glucoside);

DF = dilution factor;

ε = molar extinction coefficient (26,900 L·mol^−1^·cm^−1^ for cyanidin-3-glucoside);

l = path length (cm).

The total monomeric anthocyanin content was then calculated and expressed as micrograms of anthocyanin per gram of dry weight of each sample (μg/g dry weight).

#### 2.5.3. Determination of Total Phenolic Compounds

The total phenolic content was determined using the Folin–Ciocalteu assay, as described by Khawsuk et al. [21], with some modifications. First, gallic acid standard solutions were prepared at various concentrations (0.04–0.28 mg/mL). Crude instant rice powder extracts were prepared at various concentrations (0.2–1.0 mg/mL). Then, 5 µL of each sample or standard solution was mixed with 10 µL of Folin–Ciocalteu’s phenol reagent and incubated at room temperature for 1 min. Then, 150 µL of 5% sodium carbonate was added. The mixture was then incubated at room temperature in the dark for 1 h. Absorbance was measured at 760 nm using a spectrophotometer. Each experiment was performed in triplicates. The absorbance (OD760) of gallic acid standard solutions was measured at various concentrations to generate a standard curve, from which the linear regression equation and correlation coefficient (R2) were obtained. The OD760 values of the samples were then substituted into the linear equation of gallic acid to calculate the phenolic concentration. Phenolic concentration in the sample was then presented as milligrams of gallic acid equivalents (mg GAE)/gram dry weight.

#### 2.5.4. DPPH (1,1-Diphenyl-2-picrylhydrazyl) Radical Scavenging Assay

The radical scavenging activity of the crude instant rice powder extracts was determined using the DPPH method, following the protocol described by Khawsuk et al. [20], with some modifications. Briefly, ascorbic acid standard solutions were prepared at various concentrations (0.02–0.18 mg/mL). Crude instant rice powder extracts were prepared at various concentrations (0.2–1.4 mg/mL). Each sample (40 µL) was mixed with 200 µL of 60 µM DPPH solution in 40% acetone. The mixture was incubated at room temperature in the dark for 30 min before analysis. The absorbance was measured at 517 nm using a spectrophotometer. Each experiment was performed in triplicate using 40% acetone as a blank control. This procedure was performed using ascorbic acid as the standard, for which no calibration curve correction was applied. The percentage of radical scavenging activity was calculated using the following equation:

(7)  Scavenging Activity (%) = (absorbance_control_ − absorbance_sample_/absorbance_control_) × 100

The antioxidant capacity is represented as the half-maximal inhibitory concentration (IC_50_) value for antioxidant activity was determined as the concentration of the sample required to inhibit 50% of free radical activity.

#### 2.5.5. Ferric Reducing Antioxidant Power (FRAP) Assay

The ferric reducing power of the crude instant rice powder extracts was determined using the FRAP assay as described by Khawsuk et al. [20]. The ascorbic acid standard solutions were prepared at various concentrations (0.02–0.18 mg/mL). Crude instant rice powder extracts were prepared at various concentrations (0.2–1.4 mg/mL). Each sample (40 µL) was mixed with 100 µL of 0.2 M sodium phosphate buffer (pH 6.6) and 100 µL of 1% potassium ferricyanide solution. The mixture was then incubated at 50 °C for 20 min. The sample was then mixed with 100 µL of 10% trichloroacetic acid and centrifuged at 2200× *g* for 5 min at room temperature. The supernatant (100 µL) was collected and mixed with 200 µL of distilled water and 20 µL of 0.1% ferric chloride solution. Absorbance was measured at 700 nm. Each experiment was performed in triplicate, and the ferric reducing power of the crude instant rice powder extracts was determined as mg of ascorbic acid equivalents per gram dry weight, calculated from an ascorbic acid standard curve.

#### 2.5.6. In Vitro CaOx Crystal Formation Assay

This experiment was performed according to the method described by Ly et al. [21], with some modifications. CaOx crystals were prepared from 5 mM calcium chloride (CaCl_2_) and 7.5 mM sodium oxalate (Na_2_C_2_O_4_) in a buffer containing 0.05 mM Tris-HCl and 0.15 M sodium chloride (NaCl) at pH 6.5. The solutions were mixed and incubated in a water bath at 60 °C for 1 h, followed by overnight incubation at 37 °C. The experiment was divided into 10 groups. Group 1 served as the negative control. Group 2 served as the positive control and was treated with potassium citrate. Group 3 was treated with instant RD43 rice-powder. Group 4 was treated with instant Hom Mali 105 rice-powder. Group 5 was treated with instant RD6 glutinous rice powder. Group 6 was treated with instant Khiaw Ngoo glutinous rice. Group 7 was treated with instant Hom Mali Daeng rice powder. Group 8 was treated with instant Riceberry powder. Group 9 was treated with instant Luem Pua glutinous rice powder. Group 10 was treated with instant Kum Lanna glutinous rice powder. Each group was prepared at concentrations of 100, 200, 400, 800, 1000, 1200, and 1400 µg/mL and mixed with CaOx crystals. All groups were incubated at 37 °C for 30 min. Absorbance was measured at 620 nm using a spectrophotometer. The percentage inhibition of CaOx crystal formation was calculated using the following equation:

(8)  Inhibition (%) = (absorbance_control_ − absorbance_sample_/absorbance_control_) × 100

### 2.6. Statistical Analysis

All experiments in this study were performed in at least three replicates, and the results are expressed as means ± standard deviations. Significant differences between means were measured using analysis of variance (ANOVA), supplemented by t-tests and Duncan’s multiple range tests for post hoc comparisons. Heat map generated using TBtools software version 2.363. Principal component analysis (PCA) biplots, hierarchical cluster analysis, PCA, and k-means cluster analysis were performed using OriginPro 2022 version 9.9.0.225. Data were tested for significance at *p* < 0.05.

## 3. Results and Discussion

### 3.1. Appearance and Proximate Analysis of Brown Rice Grains

**Figure 2:**
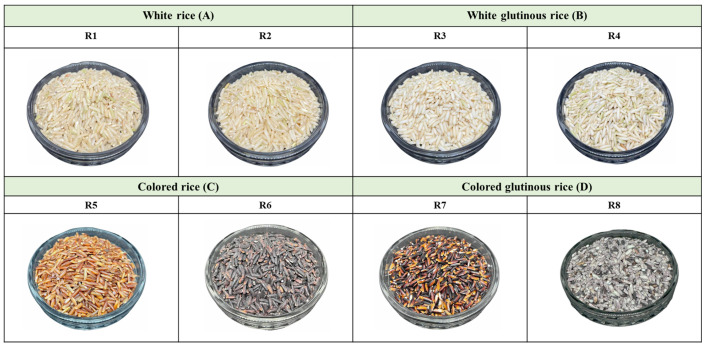
Appearance of brown rice grain.

Figure 2 shows the appearance of the eight varieties of brown rice grains. There were four groups of samples: group A: white rice (R1 and R2), group B: white glutinous rice (R3 and R4), group C: colored rice (R5 and R6), and group D: colored glutinous rice (R7 and R8). Observation of the appearance of rice grains revealed that the color of brown rice is expressed in the pericarp, which has different colors ranging from white, red, dark brown, grayish brown, and almost black-purple. Grain dimensions include the length, width, thickness, and shape of the grain. Grain size and shape are characteristics of the variety and vary depending on the variety and growing conditions. All eight rice varieties used in this study were indica, which is a long and slender grain type. In general, indica is a type of rice grown in monsoon regions of India, Sri Lanka, Thailand, Vietnam, and China. It has tall plants, soft straw, and long oval grains [22]. Most rice grown in Thailand is indica, including both glutinous and non-glutinous rice [23]. The proximate analysis of the brown rice grain is shown in Table 1. The results showed that all eight brown rice grain varieties had energy (348.74–361.19 Kcal), total carbohydrate (71.10–76.73 g), moisture (12.30–13.97 g), protein (7.58–10.93 g), fat (1.49–3.61 g), total dietary fiber (1.84–5.17 g), ash (0.72–1.55 g), and reducing sugar (0.00–0.45 g) per 100 g sample. The eight varieties of brown rice grains are mainly composed of carbohydrates. The next most abundant components were protein, fat, fiber, and ash. Rice grains generally contain approximately 12–14% moisture [24]. The eight varieties of brown rice grains have different chemical compositions and unique characteristics. The chemical composition of rice flour, particularly its protein and fat contents, strongly influences extrusion behavior. Elevated levels of these components reduce product expansion after exiting the die, resulting in a less porous structure and, consequently, lower rehydration efficiency [12]. Brown rice, which contains higher amounts of fat and dietary fiber than polished white rice, requires a lower initial moisture content and a higher barrel temperature during extrusion. Increasing the screw speed reduces the residence time, resulting in better expansion of the extrudate. Furthermore, HTST processing helps preserve heat-sensitive nutrients and bioactive compounds.

The white rice in group A, RD43, is a single-crossbreed hybrid between the Suphan Buri fragrant rice variety (mother variety) and the Suphan Buri 1 variety (father variety). RD43 rice is a large-molecule rice flour with a moderate-to-low glycemic index of approximately 57 [25]. It also has a low amylose value of 19.04%, making RD43 rice soft, easy to eat, and converting sugar more slowly, resulting in a slower rise in blood sugar levels, helping to prevent hunger, and making it suitable for diabetics [26]. Hom Mali 105 or Thai Jasmine rice is soft after cooking and has a fragrant smell similar to that of pandan leaves. The aroma of rice comes from a volatile substance called 2-acetyl-1-pyrroline, which can evaporate. To maintain the aroma of rice for a long time, it should be stored in a cool place [27]. Hom Mali 105 had an amylose value of 16.95%. RD43 and Hom Mali 105 were classified in the low-amylose (12–20%) rice group based on their amylose content. The variation in amylose content depends on the cultivar and source of amylose. The direct effect on the vegetarian component is external, personalized adhesion, and the acidity of starch digestion in the resistant starch (RS) content of food. Rice flour with a low amylose content promotes better stickiness and a softer texture. Rice flour with a high amylose content is central and crispy in food products [26]. The white glutinous rice in group B, RD6, or Kao Khor 6 was obtained by irradiating 20 kilorads of gamma radiation to induce mutations in the jasmine rice seeds, which were then grown and selected to obtain a high-yielding glutinous rice strain. RD6 has a very low amylose content of 7.33%, has good quality cooked rice, is soft and chewy, and has a fragrant smell [28]. Khiaw Ngoo is a hybrid glutinous rice that is a result of improving the breed from RD6 rice. The grain contains no more than 7% amylose [29]. The white rice in group C, Hom Mali Daeng, is a native rice variety that is a mutation of Hom Mali 105. The grains have a red pericarp and an amylose content of 8.46 [30]. Riceberry is a cross between the Hom Nin and Hom Mali 105 varieties. It has a deep purple color, long and slender grains, shiny skin, unique aroma, and sweet taste. The grain contains 16.61% amylose [31]. The white glutinous rice in group D, Leum Pua, is a native upland rice variety with a red and dark purple pericarp and low amylose content (3.36%). When cooked, it has a fragrant aroma, crunchy texture, and chewy inside of grains [32,33]. Kum Lanna or Purple Glutinous Rice is widely grown in the North and Northeast of Thailand. It has a dark red or purple-black color in the pericarp grains only and a low amylose content (0–6.20%). Rice milling is a process that removes bran and germ. The resulting rice grains are light purple-white or pink-white because the starch grains inside do not contain as much anthocyanin as the bran. However, Kum Lanna is more popularly consumed as brown rice than as white rice because the milling process causes the loss of nutrients and anthocyanins in the bran [34]. The amounts of reducing sugars in non-glutinous and glutinous rice were compared. Glutinous rice generally has a high amylopectin content and glycemic index, indicating that it can quickly raise blood sugar levels and be digested quickly [35].

### 3.2. Physicochemical Analysis of Brown Rice Grain Powder

#### 3.2.1. Appearances and Color

**Figure 3:**
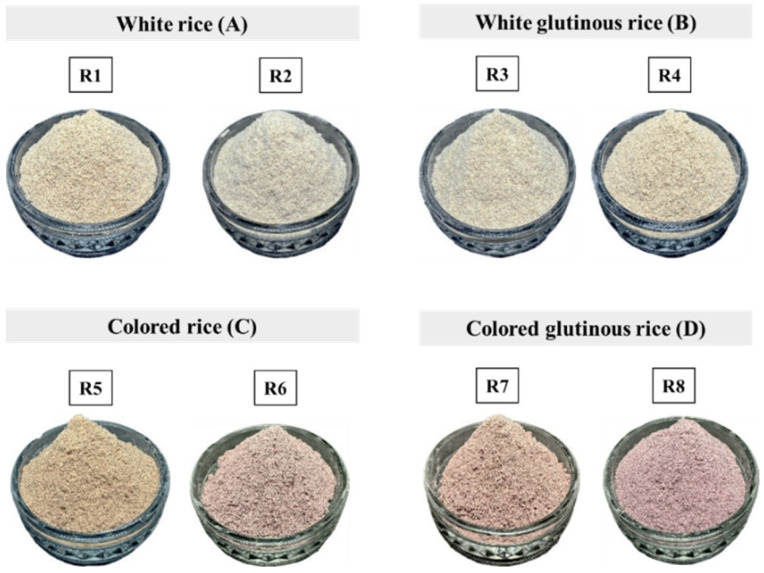
Raw rice powder samples including (R1) RD43, (R2) Hom Mali 105, (R3) RD6, (R4) Khiaw Ngoo, (R5) Hom Mali Daeng, (R6) Riceberry, (R7) Leum Pua, and (R8) Kum Lanna.

**Figure 4:**
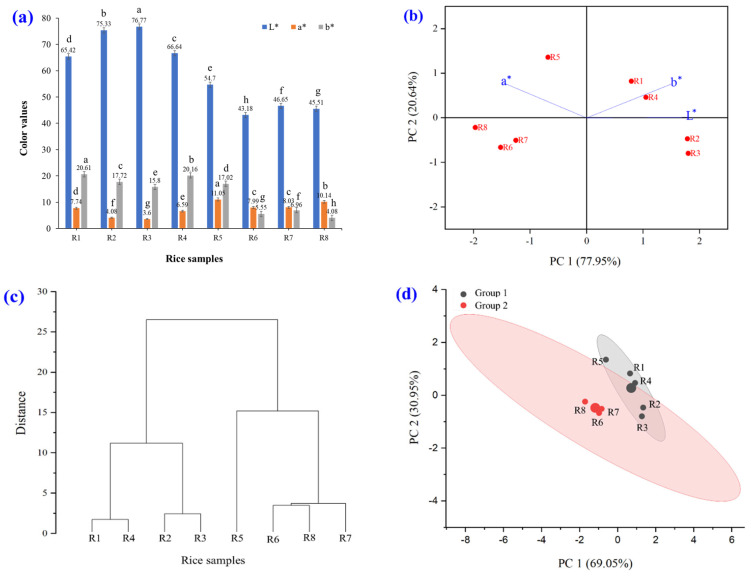
Color characteristics of raw rice powder samples including (R1) RD43, (R2) Hom Mali 105, (R3) RD6, (R4) Khiaw Ngoo, (R5) Hom Mali Daeng, (R6) Riceberry, (R7) Leum Pua, and (R8) Kum Lanna; (**a**) color value; (**b**) principal component analysis (PCA) biplot; (**c**) hierarchical cluster analysis and (**d**) PCA and k-means cluster analysis of rice color.

The appearance and color of eight brown rice varieties were analyzed after processing with an extruder and grinding into a fine powder (see Figure 3 and Figure 4). The results showed statistically significant differences in the L* values (*p* ≤ 0.05). White and white glutinous rice had higher brightness levels (65.42–76.77) than colored and colored glutinous rice (43.18–54.70). All samples exhibited positive a* values, indicating a shift toward red. Colored and glutinous rice varieties had higher a* values (7.99–11.05) than their white counterparts (3.60–7.74). The b* values, which fell within the yellow range, were also positive. Samples R1–R5 showed higher b* values (15.80–20.61) than R6–R8 (4.08–5.55), consistent with their visual appearance in Figure 3.

Principal Component analysis (PCA) biplot, where the X-axis represents Principal Component 1 (PC1) and the Y-axis represents Principal Component 2 (PC2). Each point in the graph represents the relationship between the color values of the eight varieties of brown rice grains. Points that are close together indicate rice grains with similar color values. Points that are far apart indicate rice grains with statistically different characteristics. From this PCA graph, it can be seen that PC1 is the axis that explains the most variance in the data (77.95%), which is the characteristic of the color value that is most important in this dataset. Data that are clustered close together indicate that the color values of the rice grains are similar (Figure 4b). This is related to the hierarchical cluster analysis shown in Figure 4c. The hierarchy level at the bottom of the dendrogram represents the eight varieties of brown rice grains that are not yet grouped. As one moves up the tree, similar samples cluster to form larger groups. If the linkages are close together, the rice grain samples have high color value similarity. They were divided into four subgroups: (1) R1 and R4; (2) R2 and R3; (3) R5; and (4) R6, R7, and R8. If the links are far apart, it means that the rice grain samples have high differences in color. PCA and k-means clustering were used to reduce the dimensionality to observe the trend of the data, whereas k-means was used to divide similar groups (Figure 4d). The data were divided into two large groups (Groups 1 and 2) according to the color of the brown rice grain. Points with the same color in the same group indicate that similar data are grouped in the same cluster. The largest circle indicates the centroid (center of each cluster), which is the average of the data for each group. Interpretation of the result group 1 (black) is the color of brown rice grain that is in the range of white-yellow to light-brown. Group 2 (red) represents the color of brown rice grains in the range of pink-red to purple. The main pigments found in colored brown rice grains are anthocyanins, which accumulate to give the grain its color, ranging from pink to purple-black. The anthocyanins found in colored brown rice grain are a combination of cyaniding-3-glucoside (orange-red), peonidin-3-glucoside (orange-red), peargomidin-3-glucoside (orange) and delphinidin-3-glucoside (blue-red) [36]. In general, the type and number of anthocyanins present in colored rice vary depending on genetic expression. Anthocyanins are the most important antioxidants in rice because they are the most abundant and water-soluble, making them easily absorbed by the body. Therefore, the consumption of colored brown rice can help reduce oxidative stress within cells. In addition, anthocyanins stimulate blood circulation in the arteries, especially the capillaries, and can help prevent the accumulation of clogged fat in the blood vessels [34,37].

#### 3.2.2. Moisture and a_w_

**Figure 5:**
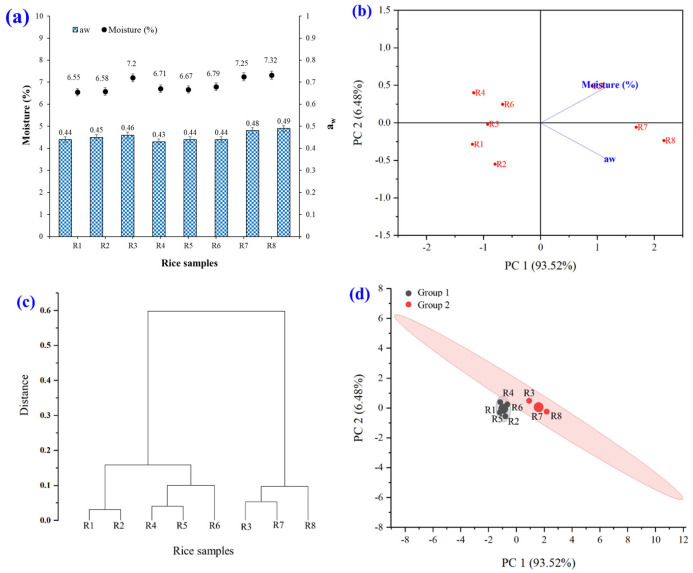
Moisture and a_w_ characteristics of raw rice powder samples including (R1) RD43, (R2) Hom Mali 105, (R3) RD6, (R4) Khiaw Ngoo, (R5) Hom Mali Daeng, (R6) Riceberry, (R7) Leum Pua, and (R8) Kum Lanna; (**a**) moisture and a_w_ value; (**b**) principal component analysis (PCA) biplot; (**c**) hierarchical cluster analysis and (**d**) PCA and k-means cluster analysis of moisture and a_w_.

The moisture and a_w_ characteristics of raw rice powder samples are shown in Figure 5. The experimental results showed that the moisture (6.55–7.32%) and aw (0.43–0.49) values were statistically different (*p* ≤ 0.05). The PCA biplot, hierarchical cluster analysis, PCA, and k-means cluster analysis of moisture and aw were investigated. The brown rice grain powder was divided into two large groups (groups 1 and 2). The interpretation of the result is that group 1 (black) comprised R1, R2, R4, R5, and R6. Group 2 (red) comprised R3, R7, and R8.

Moisture content and a_w_ in rice powder are critical determinants of its quality, shelf life, and processing characteristics. Regarding shelf life, an excessive moisture content, exceeding 13–14%, predisposes rice powder to microbial proliferation, including mold and bacteria, leading to quality degradation and rancidity [38]. Conversely, if the moisture content and a_w_ are excessively low, the rice powder may become hard, brittle, and lose its water-absorbing capacity when incorporated into food products. Optimal moisture and a_w_ levels ensure that rice powder maintains a desirable texture, resists clumping, and dissolves readily in water. Conversely, elevated moisture and aw levels can cause clumping, complicating its utilization [39,40]. Furthermore, chemical reactions, moisture content, and a_w_ influence the oxidation of fats in rice powder, potentially resulting in rancidity and accelerating enzymatic reactions that alter color or cause deterioration during extended storage. In the food industry, appropriate moisture content and aw facilitate the integration of rice powder with other ingredients, such as in bakery products or infant food. Inadequate moisture content and aw can lead to production challenges, including product extrusion or melting [38,41].

#### 3.2.3. Aroma Analysis

**Figure 6:**
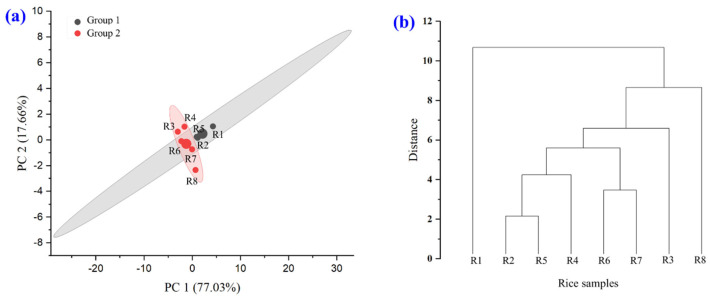
Aroma characteristics of raw rice powder samples including (R1) RD43, (R2) Hom Mali 105, (R3) RD6, (R4) Khiaw Ngoo, (R5) Hom Mali Daeng, (R6) Riceberry, (R7) Leum Pua, and (R8) Kum Lanna; (**a**) PCA and k-means cluster analysis of aroma value and (**b**) hierarchical cluster analysis.

Aroma and taste are important factors in determining the quality and characteristics of rice, as well as consumer demand. The aroma characteristics of the brown rice grain powder were analyzed using PCA. The aroma of the samples was detected using an electronic nose at room temperature. PCA and k-means cluster analysis of aroma values indicated that the samples could be categorized into two large groups (groups 1 and 2). The interpretation of the result for group 1 (black) is R1, R2, and R5. Group 2 (red) comprised R4, R6, R7, R3, and R8 (Figure 6a). In the hierarchical cluster analysis, the X-axis shows the clustered brown rice grain powder data, and the Y-axis shows the distance used to measure the similarity of the clusters (Figure 6b). Vertical lines indicate the degree to which the clusters are clustered. In the analysis of the results, small clusters were clustered before the low levels of the dendrogram (low distance values), and the closest data were clustered, such as R2 and R5. At higher clustering levels, as the distance increased, small clusters were clustered into larger clusters; for example, at a distance of approximately 6, several clusters were clustered together. The highest level of clustering, at the most distant (approximately 11), the final clustering occurs, which means that at this point, all the data are clustered into one cluster.

The aroma of each rice variety is caused by different chemicals, mostly volatile compounds found in rice bran and rice pulp. Rice with a distinct aroma from 2-acetyl-1-pyrroline (2AP) is the main compound that gives rice a fragrant aroma similar to that of pandan leaves, toast, or new rice. It is abundant in jasmine rice and rice with aroma genes. R2 is a highly fragrant cultivar with a high amount of 2AP, making the aroma distinct. The aroma is most pronounced when the rice is new and decreases with prolonged storage [27,42]. R5 is a native rice variety that mutated from R2. It has a similar aroma to that of R2 [30]. In addition, rice has a specific smell from other compounds that are not mainly caused by the aroma of 2AP, but there are volatile compounds that cause specific smells, such as phenolic compounds, aldehydes, alcohols, and fatty acids [43]. Non-aromatic red and black rice had significantly lower levels of volatile compounds than aromatic white and black rice. Aromatic black rice was found to contain high levels of both aldehydes (3-methylbutanal, 2-methylbutanal, 2-methylpropanal, pentanal, and hexanal) and alcohols (butane-2,3-diol, pentan-1-ol, hexan-1-ol). Biosynthetically, these unique volatile compounds of black glutinous rice are proposed to be formed by the degradation of branched-chain amino acids (L-leucine, L-isoleucine, and L-valine) and polyunsaturated fatty acids (linoleic acid and α-linolenic acid) by branched-chain aminotransferases, keto-acid decarboxylases, and 9-lipoxygonases and 13-lipoxygeases, respectively. The proposed amino acid and fatty acid degradation pathways are consistent with the profiles of the major volatile compounds detected in Thai native colored rice cultivars [43].

#### 3.2.4. WAI, WSI, and SP

**Figure 7:**
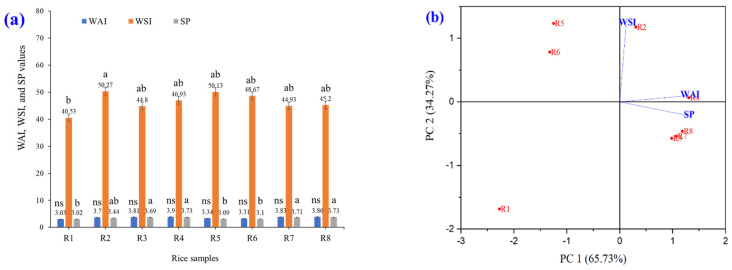

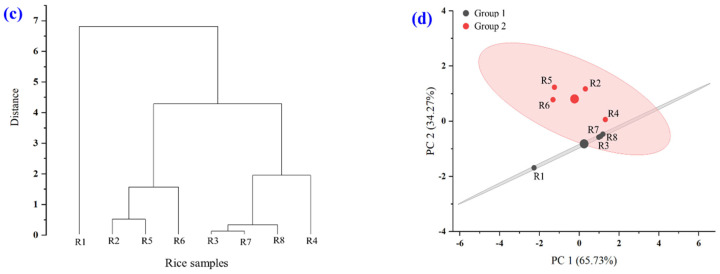
WAI, WSI, and SP of raw rice powder samples including (R1) RD43, (R2) Hom Mali 105, (R3) RD6, (R4) Khiaw Ngoo, (R5) Hom Mali Daeng, (R6) Riceberry, (R7) Leum Pua, and (R8) Kum Lanna;  (**a**) WAI, WSI, and SP; (**b**) principal component analysis (PCA) biplot; (**c**) hierarchical cluster analysis and (**d**) PCA and k-means cluster analysis of WAI, WSI, and SP value.

Eight varieties of brown rice were extruded, ground into fine powders, and subsequently analyzed for their WAI, WSI, and SP (Figure 7). The experimental results showed that the WAI and SP values were statistically significantly different (*p* ≤ 0.05). White and colored glutinous rice (R4, R4, R7, and R8) had higher WAI and SP values than white and colored non-glutinous rice (R1, R2, R5, and R6). The WSI values of the eight varieties of brown rice grains were not significantly different (*p* ≤ 0.05). From this PCA graph, it can be seen that PC1 is the axis that explains the most variance in the data (65.73%), which is the characteristic of the WAI, WSI, and SP values that are most important in the dataset. Data that are clustered close together indicate that the color values of the rice grains are similar (Figure 7b). This is related to the hierarchical cluster analysis shown in Figure 7c. The hierarchy level, the bottom of the dendrogram, represents the eight varieties of brown rice grains that are not yet grouped. As one moves up the tree, similar samples are clustered to form larger groups. If the links are close together, the rice grain samples have high WAI, WSI, and SP value similarity. They can be divided into three subgroups: (1) R1; (2) R2, R5, and R6; and (3) R3, R4, R7, and R8. In PCA and k-means clustering, PCA reduces the dimensionality to observe the trend of the data, whereas k-means is used to divide similar groups (Figure 7d). Differentiation by the WAI, WSI, and SP of brown rice grains, the data were divided into two large groups (groups 1 and 2). Points with the same WAI, WSI, and SP in the same group indicate that similar data are grouped in the same cluster. The largest circle indicates the centroid (center of each cluster), which is the average of the data for each group. The result for group 1 (black) is in the range of glutinous rice, and that for group 2 (red) is in the range of rice.

The WAI, WSI, and SP of glutinous rice were higher than those of non-glutinous rice because rice has a higher amylose content than glutinous rice flour, which makes it less able to absorb water than glutinous rice. Glutinous rice has a high amylopectin content, which helps glutinous rice flour absorb water well, is highly soluble in water upon contact with water, and glutinous rice has the ability to absorb water and expand well, causing it to expand significantly when exposed to hot water [44]. The WAI is a measure of a flour’s ability to absorb water when mixed with water over time. When flour is placed in water, it absorbs water into its structure, thereby increasing the gel volume. WAI indicates the amount of water absorbed relative to the flour weight. Swelling and water absorption cause flour to expand or swell, and the WAI measurement reflects this expansion ability. WAI values are important for evaluating flour properties in food manufacturing, particularly in products requiring viscosity and swelling [45]. When starch is boiled in excessive H_2_O, the H bonds break, destroying the crystalline form of the starch. The H_2_O molecules form H bonds with the -OH groups of the amylose/amylopectin in contact with the starch, increasing its solubility and swelling capacity. The solubility and swelling intensity indicate the degree of cohesion between starch sequences. The extent of this cohesion is controlled by the conformation of amylose or amylopectin, whereas the tendency of starch to swell depends primarily on the structure of amylopectin. Glutinous rice flour has a higher swelling strength than that of non-glutinous rice flour [46].

#### 3.2.5. Viscosity

**Figure 8:**
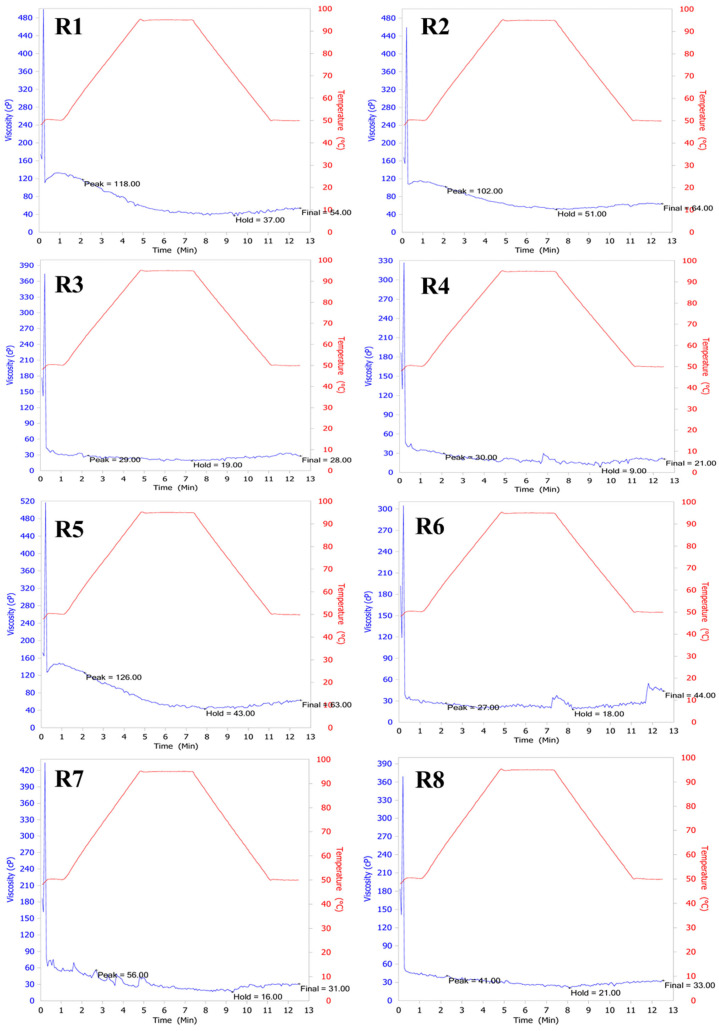
Viscosity measurement using a Rapid Visco Analyzer of raw rice powder samples including (R1) RD43, (R2) Hom Mali 105, (R3) RD6, (R4) Khiaw Ngoo, (R5) Hom Mali Daeng, (R6) Riceberry, (R7) Leum Pua, and (R8) Kum Lanna.

The high-temperature extrusion process induces partial dextrinization of starch molecules, resulting in smaller fragments, lower peak viscosity, and reduced stickiness due to the disruption of long-chain starch structures [12]. Nevertheless, extrusion produces a well-dissolved and uniform rice powder. The pasting profiles of extruded rice powders varied between rice varieties, reflecting the combined effects of starch composition and extrusion parameters on their functional properties (Figure 8). The powder samples from the glutinous rice family, R3 and R4, exhibited pasting properties characterized by low peak viscosity (29–30 cP), minimal breakdown values (10 cP), and low setback viscosity (9 cP), indicating a stable starch granule structure under heat and shear stress. In contrast, samples of white rice and non-glutinous colored rice, such as R1, R2, and R5, had high peak viscosity values (102–126 cP) and high breakdown values (51–83 cP), indicating high swelling values. Similarly, white glutinous rice, such as R6, had low peak viscosity values (27 cP) but high setback viscosity values (26 cP), indicating a strong retrogradation tendency. Colored glutinous rice, such as R7 and R8, had moderate PV values (41–56 cP), breakdown values (20–40 cP), and relatively low setback viscosity values (12–15 cP).

The observed decrease in peak viscosity after extrusion was consistent with previous reports, which attribute viscosity reduction to starch degradation and dextrinization under high-temperature and high-shear conditions. Huang et al. [47] provided a mechanistic review showing that extrusion preferentially cleaves amylopectin branch points, inducing order–disorder transitions and thereby lowering molecular weight and reducing pasting viscosities. Similarly, Liu et al. [11] reported significantly lower peak, trough, breakdown, and final viscosity values in extruded rice starch and flour compared to native starch, which aligns well with our RVA observations. These findings collectively confirm that the reduction in viscosity parameters in this study agrees well with previous observations on the effects of extrusion on starch functionality.

Amylose and amylopectin are the main variables affecting the viscosity of starch. Other factors include chemical composition, gelatinization temperature, water content, pH, time, rice type (variety), additives, mixing speed, and processing conditions [48]. Extrusion is a process that alters the physical structure of rice starch by applying heat and high pressure. This affects amylose and amylopectin in starch, causing it to expand and change its physical properties. The extrusion of starch through the extruder affects the viscosity of different rice varieties in different ways, depending on the starch properties and the varying amylose and amylopectin structures [49]. The interaction between protein and fat with amylose is another factor affecting the viscosity of rice powder. Amylose is a polysaccharide composed of long glucose chains with a flexible linear structure. Amylose interacts with proteins and lipids to form stronger pasty structures. Amylose–lipid interactions, such as amylose binding to lipids, increase the viscosity of the dough by preventing expansion and solubility [50]. Amylose–protein interactions: Amylose can interact with proteins in various ways, such as chemically binding or physically binding through hydrogen bonds, polar interactions, and ionic interactions. Proteins help to form a stronger pasty structure. Proteins can contribute to the viscosity of dough by increasing its elasticity and water-binding capacity [51].

The distinct structural roles of amylose and amylopectin in starch granules mechanistically explain these results. Amylose, a mostly linear polymer, forms compact helical complexes that restrict water penetration and swelling, leading to lower peak viscosity after extrusion. In contrast, highly branched amylopectin promotes granule swelling and viscosity development but is more prone to molecular degradation under heat and shear. During extrusion, the combined effects of heat, pressure, and shear disrupt amylopectin branches and partially gelatinize starch, reducing molecular weight and viscosity. Consequently, rice varieties with higher amylopectin content (e.g., glutinous types) exhibit lower viscosity stability and greater sensitivity to extrusion-induced degradation, whereas those with higher amylose content maintain more stable viscosity behavior [52].

### 3.3. Bioactive Properties of Brown Rice Grain Powder

#### 3.3.1. Antioxidant Activities

The extraction yield, total monomeric anthocyanin, and total phenolic compounds of the crude instant rice powder extracts are shown in Table 2. Overall, the RD43 sample exhibited a moderate extraction yield (1.84%), low anthocyanin content (0.03 ± 0.00 μg/g dry weight), and moderate phenolic content (107.20 ± 1.25 mg GAE/g dry weight). Hom Mali 105 and RD6 exhibited relatively low extraction yields (1.63–1.67%), anthocyanin content (0.01 ± 0.00 μg/g dry weight), and phenolic content (65.83 ± 1.26 and 62.72 ± 1.06 mg GAE/g dry weight, respectively). Khiaw Ngoo had the highest extraction yield (2.72%), although anthocyanin was very low (0.01 ± 0.00 μg/g dry weight). The phenolic content was relatively high (120.80 ± 1.77 mg GAE/g dry weight). Hom Mali Daeng showed the lowest extraction yield (1.57%) and anthocyanin level (0.01 ± 0.00 μg/g dry weight); however, a high phenolic content (131.23 ± 2.62 mg GAE/g dry weight) was observed. The extraction yield of Riceberry was low (1.57%), but both anthocyanin (0.01 ± 0.00 μg/g dry weight) and phenolic compound (161.43 ± 4.35 mg GAE/g dry weight) contents were higher than those of white pigmented rice cultivars. Luem Pua showed a high extraction yield (2.32%), high anthocyanin content (0.06 ± 0.01 μg/g dry weight), and the highest total phenolic content (249.72 ± 4.95 mg GAE/g dry weight). Kum Lanna exhibited a low extraction yield (1.65%) but showed the highest anthocyanin content (0.19 ± 0.01 μg/g dry weight) and a high phenolic level (196.49 ± 2.15 mg GAE/g dry weight).

The antioxidant activity of crude instant rice powder extracts, as assessed by DPPH radical scavenging (IC50) and FRAP assays, is detailed in Table 3. The results varied significantly between the rice cultivars. In the DPPH assay, a lower IC50 value signifies higher free-radical scavenging activity. The standard antioxidant, ascorbic acid, demonstrated the highest activity (IC50 = 0.06 ± 0.00 mg/mL). Colored rice cultivars generally exhibited greater activity compared to non-colored ones. Kum Lanna displayed the highest DPPH radical scavenging activity with the lowest IC50 value (0.31 ± 0.0 mg/mL), followed by Luem Pua (0.42 ± 0.02 mg/mL), Riceberry (0.48 ± 0.02 mg/mL), and Hom Mali Daeng (0.49 ± 0.01 mg/mL). Conversely, white rice cultivars such as Hom Mali 105, RD6, and Khiaw Ngoo exhibited lower activity, with IC50 values ranging from 1.43 to 2.00 mg/mL. The results of the FRAP assay were consistent with those of the DPPH assay, with colored rice cultivars demonstrating high ferric reducing power. Luem Pua recorded the highest FRAP value (9.88 ± 0.00 mg ascorbic acid equivalents/g dry weight), followed by Riceberry (7.78 ± 0.02 mg ascorbic acid equivalents/g dry weight), Kum Lanna (5.74 ± 0.16 mg ascorbic acid equivalents/g dry weight), and Hom Mali Daeng (5.46 ± 0.15 mg ascorbic acid equivalents/g dry weight). In contrast, non-colored rice, RD43, exhibited very low reducing power (1.22 ± 0.15 mg ascorbic acid/g dry weight). Interestingly, other non-colored rice, including Hom Mali 105, RD6, and Khiaw Ngoo, exhibited no detectable reducing power activity.

Anthocyanins are phenolic compounds and a subgroup of flavonoids responsible for the red, purple, and blue hues in rice. These compounds exhibit antioxidant properties and contribute to the prevention of cellular degeneration. Antioxidants are agents that mitigate the presence of free radicals in the body. Free radicals are molecules characterized by unpaired electrons, which can inflict damage on cells and DNA when present in excessive quantities [53]. The experimental findings align with those documented by Kammapana [54]. The study examined the anthocyanin and phenolic compound content, as well as the antioxidant activities, of various rice varieties. Black, purple, and red rice are noted for their high levels of phenolic compounds and anthocyanins. Furthermore, rice varieties with elevated levels of anthocyanins and phenolic compounds demonstrate significant antioxidant activity, with black and purple rice varieties exhibiting particularly strong antioxidant properties. Colombo et al. [53] investigated the color, phenolic profile, and antioxidant activity of Asian rice, noting that black and purple rice contain substantial levels of anthocyanins. These compounds undergo changes during processing through methods such as cooking, grinding, and drying. The study concluded that processing may slightly diminish the levels of anthocyanins, phenolic compounds, and antioxidant activity in certain rice varieties; however, sufficient concentrations are retained to confer health benefits.

#### 3.3.2. Anti-CaOx Crystal Formation

**Figure 9:**
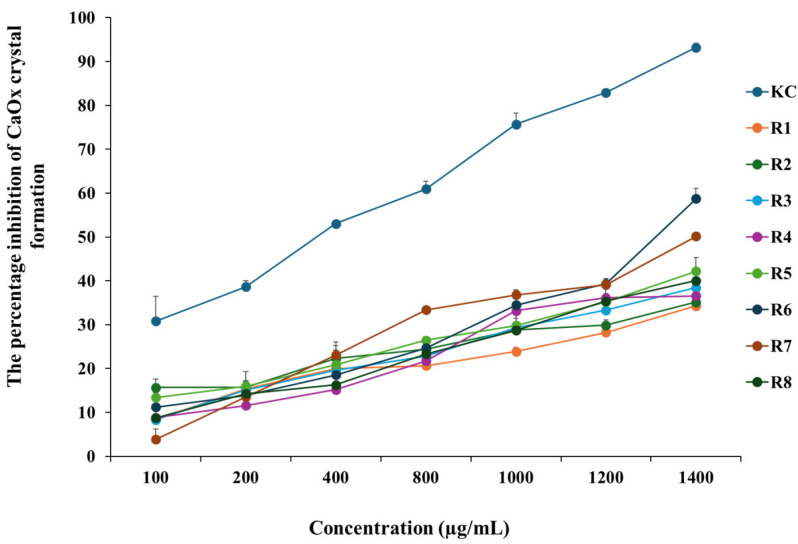
The percentage inhibition of CaOx crystal formation of 8 instant rice cultivars at the concentration of 100–1400 µg/mL compared with the positive control (KC group). Note: (R1) RD43, (R2) Hom Mali 105, (R3) RD6, (R4) Khiaw Ngoo, (R5) Hom Mali Daeng, (R6) Riceberry, (R7) Leum Pua, and (R8) Kum Lanna.

The percentage inhibition of CaOx crystal formation by instant rice powder is presented in Figure 9. The positive control (KC) demonstrated the highest percentage of inhibition, reaching 93.20% at a concentration of 1400 μg/mL. All rice cultivars exhibited a relatively increased percentage of inhibition in a dose-dependent manner. The percentage inhibition for the four colored rice cultivars, namely Riceberry, Luem Pua, Hom Mali Daeng, and Kum Lanna, was 58.71%, 50.17%, 42.23%, and 40.03%, respectively, at a concentration of 1400 μg/mL. At the same concentration, non-colored rice cultivars, including RD43, RD6, Hom Mali 105, and Khiaw Ngoo, displayed a lower percentage of inhibition, ranging from 34% to 38%.

In vitro CaOx crystal formation assays examine the development of CaOx crystals, which are associated with the propensity to form kidney stones. Typically, when these crystals accumulate and cannot be excreted from the body, they accumulate in the kidneys and form kidney stones [55,56]. Khawsuk et al. [57] investigated the antioxidant properties of Riceberry extract, an unpolished rice variety, and its capacity to inhibit the growth and aggregation of CaOx crystals, a primary step in kidney stone development. The antioxidants present in Riceberry rice were found to inhibit the growth and aggregation of CaOx crystals, potentially reducing the risk of kidney stones by decreasing the likelihood of crystal accumulation and stone formation in the urinary tract. Furthermore, black glutinous rice bran extract has been reported to inhibit the formation of CaOx crystals and reduce their aggregation. Suggesting that, the antioxidants in colored rice might have reducing properties that can chelate calcium ions. As a result, calcium ions are prevented from binding to oxalate ions and forming calcium oxalate (CaOx) crystals [57]. Several studies have demonstrated that various bioactive substances derived from diverse plants can inhibit the formation of kidney stones. These include phenolic acids like caffeic acid, ferulic acid, gallic acid, and rosmarinic acid; flavonoids like apigenin, catechin, epicatechin, rutin, and quercetin; and terpenoids, furanochromes, carotenoids, alkaloids, vitamin E, fatty acids [58], and anthocyanins [57]. The present study demonstrated that colored rice cultivars exhibited a higher inhibitory effect on CaOx crystal formation than non-colored rice cultivars. However, further studies are required to elucidate the bioactive compounds underlying the mechanisms involved in kidney stone formation.

Black glutinous rice bran extract may serve as a safe and effective alternative for mitigating the risk of kidney stones, as it is associated with minimal side effects [18]. Colored rice may be utilized in the development of future foods or dietary supplements aimed at preventing kidney stones.

### 3.4. PCA and Heatmap Analysis

**Figure 10:**
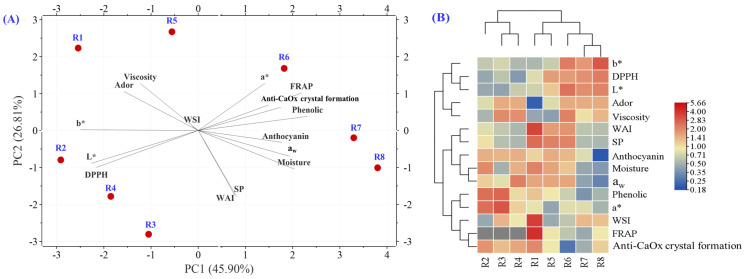
PCA biplot (**A**) and heatmap (**B**) for the physicochemical and bioactive parameters of different raw rice powder samples. Note: (R1) RD43, (R2) Hom Mali 105, (R3) RD6, (R4) Khiaw Ngoo, (R5) Hom Mali Daeng, (R6) Riceberry, (R7) Leum Pua, and (R8) Kum Lanna, b*—yellowness value, DPPH—DPPH radical scavenging assay, L*—lightness value, Ador—aroma characteristics, Viscosity—peak viscosity, WAI—water absorption index, SP—swelling power, Anthocyanin—total monomeric anthocyanin, Moisture—moisture content, a_w_—water activity, Phenolic—total phenolic compounds, a*—redness value, WSI—water solubility index, FRAP—ferric reducing antioxidant power assay, anti-CaOx crystal formation—in vitro calcium oxalate crystal formation assay.

The PCA biplot and heatmap of the physicochemical and bioactive parameters of various raw rice powder samples are presented in Figure 10. The relationships between the quality parameters of these samples were analyzed, with PC1 and PC2 accounting for 45.90% and 26.81% of the total variance, respectively. Collectively, these two axes explain approximately 72.71% of the total data, which is considered adequate for assessing the group structure. The eight rice samples, represented by red dots, that are in close proximity exhibit similar properties. It is evident that each group is distinctly separated, indicating that each type of extruded rice possesses unique physicochemical properties and biological activities. The length of the variable line significantly influences group separation, and the direction of the arrows corresponds to the PC1 and PC2 axes. These variations are clearly depicted in the heatmap’s color progression from blue to warmer tones. The samples were categorized into two groups: (1) rice R1, R2, R3, and R4, and (2) sticky rice R5, R6, R7, and R8. In terms of quality, the samples were divided into two primary groups: the first group included b*, DPPH, L*, odor, and viscosity, while the second group comprised WAI, SP, anthocyanin, moisture, aw, phenolic, a*, WSI, FRAP, and anti-CaOx crystal formation. Obviously, the data obtained from PCA biplot and heatmap indicated the relationship between rice and sticky rice powder in various dimensions such as texture, color, viscosity, ador, and important bioactive compounds resulting from different chemical compositions which vary between rice varieties as described previously.

## 4. Conclusions

Extrusion is a suitable technique for producing ready-to-use rice powder. The eight rice powder samples utilized in this study exhibited variations in their physicochemical properties, including color value, moisture content, water activity (aw), aroma, water absorption index (WAI), water solubility index (WSI), swelling power (SP), and viscosity, as well as in their biological activities, such as total monomeric anthocyanins, total phenolic compounds, antioxidant properties, and anti-CaOx crystal formation effects. Each rice variety possesses a distinct chemical composition that influences its quality. Glutinous rice powder demonstrates greater solubility in water compared to regular rice powder. Additionally, colored rice contains higher levels of bioactive compounds and exhibits more pronounced biological activities than white rice. Extruded rice powder can serve as a raw material for the development of ready-to-eat products that minimize cooking time. Flavor enhancements or nutritional fortification with additional ingredients can be incorporated to provide consumers with a wider range of options. The observed variations in the powder samples can be attributed to both the intrinsic properties of the rice varieties and the extrusion process. The yield of extruded rice powder was comparable between samples, with only a slight reduction primarily due to moisture loss during processing. Because extrusion is a nearly closed and continuous process, product losses are minimal. Furthermore, the process did not require any additive or carrier agent, unlike spray or foam-mat drying, ensuring that the obtained powder directly reflected the original rice composition. Therefore, the compositional differences between the powders are mainly related to the inherent characteristics of each rice variety, whereas the physicochemical and functional modifications (e.g., gelatinization and solubility) are primarily driven by the extrusion process. Further investigations should include the measurement of dispersion and bulk density to provide a more comprehensive understanding of the physical behavior of extruded rice powder. Research and development efforts should also focus on creating nutritionally rich dietary supplements, promoting the utilization of rice powder in local cuisine, conducting market analysis, and developing rice powder products with specific functional properties. Furthermore, resistant starch content should be measured in future studies to validate its potential effect on lowering glycemic response.

## Figures and Tables

**Table 1:** Proximate analysis of brown rice grain.

Chemical Composition (per 100 g Brown Rice)	Rice Samples							
	R1	R2	R3	R4	R5	R6	R7	R8
Moisture (g)	12.53 ± 0.10 cd	12.41 ± 0.11 d	12.30 ± 0.14 d	13.45 ± 0.21 b	13.32 ± 0.22 b	12.76 ± 0.20 b	13.24 ± 0.10 b	13.97 ± 0.20 a
Protein (g)	9.48 ± 0.17 b	8.96 ± 0.21 c	8.36 ± 0.15 d	7.58 ± 0.15 e	8.89 ± 0.21 c	9.44 ± 0.29 b	10.93 ± 0.26 a	8.37 ± 0.23 d
Fat (g)	3.27 ± 0.14 abc	2.96 ± 0.24 c	2.95 ± 0.21 c	1.49 ± 0.17 d	3.35 ± 0.25 ab	3.61 ± 0.26 a	3.18 ± 0.15 bc	1.50 ± 0.19 d
Ash (g)	1.26 ± 0.18 a	1.22 ± 0.18 a	1.43 ± 0.22 a	0.75 ± 0.17 b	1.23 ± 0.21 a	1.46 ± 0.17 a	1.55 ± 0.12 a	0.72 ± 0.23 b
Total carbohydrate (g)	71.10 ± 0.13 d	72.17 ± 0.16 c	72.16 ± 0.27 c	73.93 ± 0.21 a	70.70 ± 0.17 e	67.56 ± 0.24 g	68.56 ± 0.11 f	73.60 ± 0.14 b
Total dietary fiber (g)	2.35 ± 0.25 c	2.28 ± 0.17 c	2.79 ± 0.22 b	2.80 ± 0.10 b	2.50 ± 0.21 bc	5.17 ± 0.18 a	2.54 ± 0.26 bc	1.84 ± 0.25 d
Reducing sugar (g)	0.34 ± 0.20 a	<0.26 ± 0.24 ab	<0.26 ± 0.13 ab	0.00 ± 0.01 b	<0.26 ± 0.12 ab	<0.26 ± 0.15 ab	0.36 ± 0.12 a	0.45 ± 0.21 a
Energy (Kcal)	361.19 ± 0.18 a	360.28 ± 0.29 b	359.83 ± 0.31 b	350.65 ± 0.24 e	358.55 ± 0.32 c	361.17 ± 0.25 a	356.74 ± 0.23 d	348.74 ± 0.31 f

Mean ± standard deviation. Different letters indicate a significant difference (*p* ≤ 0.05).

**Table 2:** Extraction yield, total monomeric anthocyanin, and total phenolic compounds of crude instant rice powder extracts.

Rice Samples	Extraction Yield (%)	Total Monomeric Anthocyanin (µg/g Dry Weight)	Total Phenolic Compounds (mg GAE/g Dry Weight)
R1	1.84	0.03 ± 0.00 c	107.20 ± 1.25 g
R2	1.67	0.01 ± 0.00 d	65.83 ± 1.26 g
R3	1.63	0.01 ± 0.00 cd	62.72 ± 1.06 f
R4	2.72	0.01 ± 0.00 d	120.80 ± 1.77 e
R5	1.57	0.01 ± 0.00 d	131.23 ± 2.62 d
R6	1.57	0.01 ± 0.00 cd	161.43 ± 4.35 c
R7	2.32	0.06 ± 0.01 b	249.72 ± 4.95 a
R8	1.65	0.19 ± 0.01 a	196.49 ± 2.15 b

Mean ± standard deviation. Different letters indicate a significant difference (*p* ≤ 0.05).

**Table 3:** The DPPH radical scavenging and FRAP assays of crude instant rice powder extracts.

Rice Samples	DPPH Assay (IC_50_ mg/mL)	FRAP Assay (mg Ascorbic Acid Equivalents/g Dry Weight)
R1	1.15 ± 0.01 d	1.22 ± 0.15 d
R2	1.81 ± 0.10 b	ND
R3	1.43 ± 0.02 c	ND
R4	2.00 ± 0.10 a	ND
R5	0.49 ± 0.01 e	5.46 ± 0.15 c
R6	0.48 ± 0.02 ef	7.78 ± 0.02 b
R7	0.42 ± 0.02 ef	9.88 ± 0.00 a
R8	0.31 ± 0.00 g	5.74 ± 0.16 c

ND = non-detectable. Mean ± standard deviation. Different letters indicate a significant difference (*p* ≤ 0.05).

## Data Availability

The original contributions presented in the study are included in the article. Further inquiries can be directed to the corresponding authors.

## Abbreviations

ANOVA	Analysis of variance
a_w_	Water activity
CaCl_2_	Calcium chloride
CaOx	Calcium oxalate
D	Barrel bore
DNA	Deoxyribonucleic acid
DPPH	1,1-diphenyl-2-picrylhydrazyl radical scavenging assay
FRAP	Ferric reducing antioxidant power assay
GAE	Gallic acid equivalent
HTST	High temperature short time
IC_50_	Inhibitory concentration
KC	Positive control
L	Barrel length
Na_2_C_2_O_4_	Sodium oxalate
NaCl	Sodium chloride
PCA	Principal component analysis
R1	RD43
R2	Hom Mali 105
R3	RD6
R4	Khiaw Ngoo
R5	Hom Mali Daeng
R6	Riceberry
R7	Leum Pua
R8	Kum Lanna
RS	Resistant starch
RVA	Rapid visco analyzer
RVU	Rapid viscosity unit
SP	Swelling power
WAI	Water absorption index
Wg	The weight of the gel
Ws	The initial weight of the sample
WSI	Water solubility index
Wsl	The weight of solids obtained from the supernatant
